# Procedural Learning and Memory Rehabilitation in Korsakoff’s Syndrome - a Review of the Literature

**DOI:** 10.1007/s11065-015-9288-7

**Published:** 2015-06-06

**Authors:** Erik Oudman, Tanja C. W. Nijboer, Albert Postma, Jan W. Wijnia, Stefan Van der Stigchel

**Affiliations:** Department of Experimental Psychology, Helmholtz Institute, Utrecht University, Heidelberglaan 1, 3584 CS Utrecht, The Netherlands; Slingedael Korsakoff Center, Slinge 901, 3086 EZ Rotterdam, The Netherlands; Brain Center Rudolf Magnus, and Center of Excellence for Rehabilitation Medicine, University Medical Center Utrecht and De Hoogstraat Rehabilitation, Utrecht, The Netherlands; Department of Neurology, Brain Center Rudolf Magnus, University Medical Center, Utrecht, The Netherlands

**Keywords:** Procedural learning, Procedural memory, Korsakoff syndrome, Amnesia, Cognitive rehabilitation, Alcoholism

## Abstract

Korsakoff’s syndrome (KS) is a chronic neuropsychiatric disorder caused by alcohol abuse and thiamine deficiency. Patients with KS show restricted autonomy due to their severe declarative amnesia and executive disorders. Recently, it has been suggested that procedural learning and memory are relatively preserved in KS and can effectively support autonomy in KS. In the present review we describe the available evidence on procedural learning and memory in KS and highlight advances in memory rehabilitation that have been demonstrated to support procedural memory. The specific purpose of this review was to increase insights in the available tools for successful memory rehabilitation and give suggestions how to apply these tools in clinical practice to increase procedural learning in KS. Current evidence suggests that when memory rehabilitation is adjusted to the specific needs of KS patients, this will increase their ability to learn procedures and their typically compromised autonomy gets enhanced.

## Introduction

**Fig. 1:**
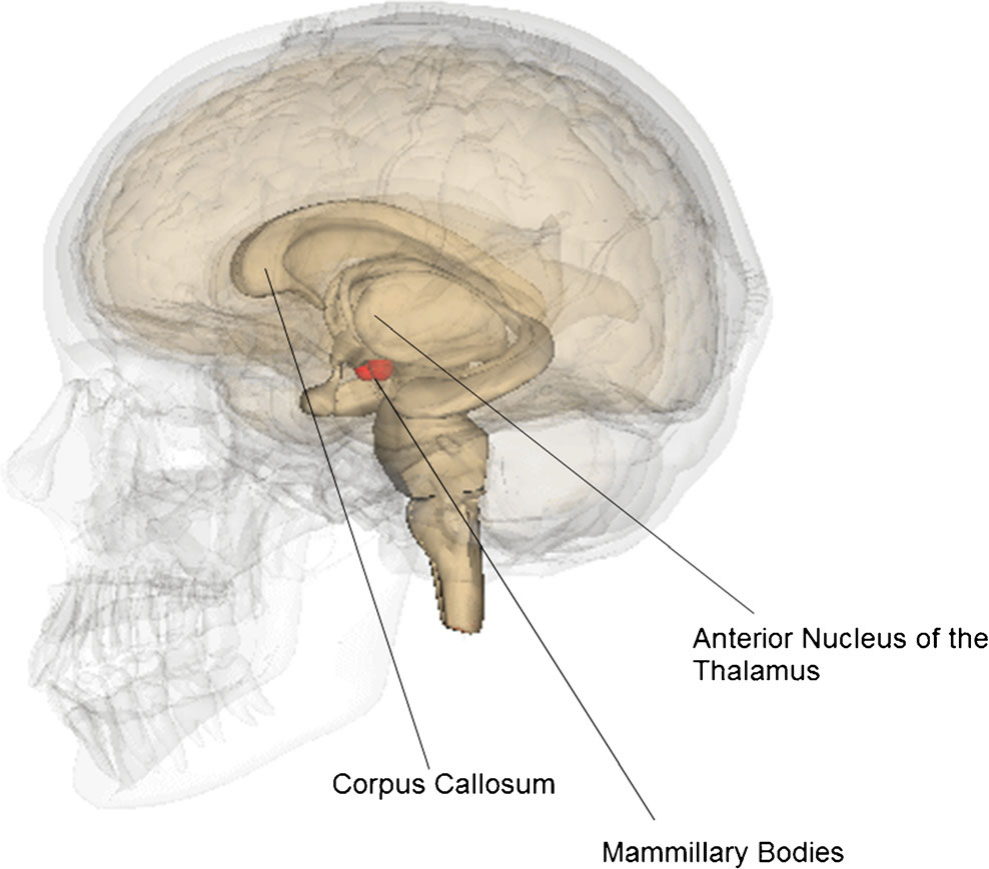
Neuronal loss in the Mammillary Bodies, the Corpus Callosum and the Anterior Nucleus of the Thalamus is common in patients with Korsakoff’s syndrome. Modified and reprinted from Anatomography under Creative Commons Attribution

Korsakoff’s syndrome (KS) is a chronic neuropsychiatric disorder that is caused by thiamine deficiency. In the industrialized world, the most common cause of thiamine deficiency is alcoholism, with around 90 % of the deficiencies associated with alcohol abuse (Harper et al. 1986; Thomson et al. 2002; Kopelman et al. 2009). Interestingly, around 15 % of the chronic alcoholics have neurological signs of KS (Kril and Harper 2012). The most essential symptom of KS is a profound declarative memory impairment for learning and remembering new material (anterograde amnesia). In KS, there is also a temporally-graded memory deficits for remote memory (retrograde amnesia) which characteristically extends back many years or decades (Kopelman et al. 1999). Commonly, executive deficits are present, such as problems with inhibition of behavior, high interference of information sensitivity, poor judgment, poor planning abilities, problem solving inabilities, and perseverative responses (Van der Stigchel et al. 2012; Oscar-Berman 2012). The cognitive problems in KS are caused by diencephalic atrophy of the brain, with damage to the anterior nucleus of the thalamus, the mammillary bodies and the corpus callosum as the most common features of KS that are not caused by the direct neurotoxic effects of alcohol (see Fig. 1 for the anatomical localization of the most common brain abnormalities in KS). (Paller et al. 1997; Kopelman 1995; Kopelman 2002; Sullivan and Pfefferbaum 2009; Kril and Harper 2012; Pitel et al. 2012; Jung et al. 2012).

KS is usually preceded by an acute neurological condition called Wernicke’s Encephalopathy, although in some cases KS seems to develop insidiously. Recent studies suggest that the most common symptom of Wernicke’s Encephalopathy is the change in mental status, frequently presenting itself as a delirium (Wang and Hazell 2010; Wijnia and Oudman 2013). Strikingly, in the acute phase memory problems are not necessarily present but will develop over the course of the syndrome (Isenberg-Grzeda et al. 2012). Severe cognitive problems are often the direct consequence of undertreated thiamine deficiency (Oscar-Berman et al. 1982; Sechi and Serra 2007; Oudman et al. 2014). In the chronic phase of KS, cognitive problems do not respond to thiamine therapy any more (Smith and Hilman 1999). The diagnosis of KS requires extensive neuropsychological and neuropsychiatric examination to establish whether explicit memory impairment is disproportionate and can only be made with certainty after at least 6 weeks of sobriety, but could effectively require more time when serious somatic conditions are present (Kopelman 1995; Day et al. 2004).

Memory difficulties are a defining characteristic of KS (Kopelman et al. 2009). Currently, it is clear that memory difficulties in KS are most profound in declarative memory (“knowing what”), while procedural learning (“knowing how”) is more preserved in KS (Squire 2004; Oudman et al. 2011). The acquisition of a skill or procedure occurs over the course of practice and is mainly thought to be nondeclarative. Because of the severity of the problems regarding “knowing what” in KS patients, many patients are in need of lifelong care (Oudman et al. 2013; Gerridzen and Goossensen 2014). Despite the severe problems it is intriguing to see that patients with amnesia sometimes show preserved learning abilities for learning skills and procedures (“knowing how”). In the literature, it is debated, both from a clinical and experimental perspective, whether procedural memory is fully spared or weakened (Hayes et al. 2012). From a clinical perspective, residual procedural learning would be relevant to the memory rehabilitation of individuals with KS, since this would indicate that patients with severe amnesia still can learn and rehabilitate in specific instances, and as such it might reduce their functional disability (Svanberg and Evans 2013; Horton et al. 2014). In the first part of the present review we detail the available evidence on motoric and cognitive procedural learning and memory in KS. In the second part we highlight the advances in memory rehabilitation which have been found to support procedural memory. Finally, we discuss how memory rehabilitation techniques can specifically support procedural learning in KS.

## Procedural Learning in Korsakoff’s Syndrome

### Motoric Procedural Learning in Korsakoff’s Syndrome

The most typical example of procedural learning is motoric procedural learning or motor skill learning. The tradition to study motoric memory and learning in KS at least partially stemmed from investigations of the bilateral hippocampal patient H.M. who was still able to learn simple motoric tasks, despite his global amnesia (Scoville and Milner 1957). In his book “Deranged memory”, George Talland (1965) defined the general aspects of procedural memory in KS. He was one of the first to note that also patients with KS are able to quickly learn a simple repetitive motor-task, such as how to use a plunger device to pick up small beads. Ever since this discovery, several paradigms have been adopted to assess motoric procedural learning in KS with variable outcomes throughout the long history of this field of research.

#### Rotor Pursuit and Mirror Drawing

In the early days of studying motoric procedural learning the most applied paradigm was the pursuit rotor task. In a typical pursuit rotor task the participant was asked to maintain contact between a stylus and a metallic target on a moving turntable, while the turntable moved 45 rounds a minute. The first experiment on motoric procedural learning in KS was performed by Cermak et al. (1973). In their study, nine patients with KS and nine controls were fully able to learn and maintain the pursuit rotor task, suggesting that this form of motoric procedural learning is preserved in KS. The authors reasoned that pursuit rotor learning in KS is intact because there are no verbally mediated choices in this task whereas patients with KS would have large impairments on tasks that require verbally mediated choices. Despite the age of this paper, it is one of the few papers on motoric procedural learning in KS that included a group of healthy controls and it is therefore still relevant to the study of motoric procedural learning. Three years later, an experiment by Brooks and Baddeley (1976) showed complementary results to the Cermak study (1973). Importantly, Brooks and Baddeley (1976) additionally showed that pursuit rotor learning was still preserved after 1 week without practice. Later, Heindel et al. (1988) also demonstrated intact pursuit rotor learning in their amnesic group compared to patients with Huntington’s disease who were severely impaired on the task. This study suggested a critical involvement of the basal ganglia in motoric procedural learning, since Huntington’s disease is associated with basal ganglia damage whereas KS is not. This finding was later elaborated on by McEntee, Mair and Langlais (1987). Their study focused on the involvement of neurotransmitters in motoric learning by means of a rotor pursuit task and a mirror tracing task. In the mirror tracing task patients had to trace the outline of an object, a six-pointed star, while looking into a mirror. After the experiment, which showed increased performance, the cerebrospinal fluid of the patients was verified for levels of norepiphrine, serotonine and dopamine metabolites. Task performance on two tasks correlated moderately positively with the dopamine metabolite (HVA), suggesting a dopamine involvement to motoric procedural learning in the mirror drawing and the pursuit task. In conclusion, studies on rotor pursuit tasks suggest that this specific form of motoric procedural learning is fully spared in KS.

#### Serial Reaction Time and Recent Motoric Tasks

In the late 80s of the last century, a different paradigm became popular, namely the serial reaction time paradigm. In a classical serial reaction time paradigm participants are seated facing a monitor and a response board below the monitor. On the response board four keys are arranged in a row. Participants are asked to press the key that is below the location in which an asterisk appears one of four locations on the monitor. A sequence is repeated throughout the experiment and reaction times on elements of this sequence are compared with reaction times to random elements. In contrast to the pursuit rotor tasks, the serial reaction paradigm does not require fine motor skills that could possibly hamper task performance on the pursuit rotor task. Also, the serial reaction paradigm makes it possible to measure reaction times as well as task accuracy. Recently, the serial reaction time paradigm has been put forward as a procedural task in three meta-analyses (Lum et al. 2014; Clark et al. 2014; Foti et al. 2015). An important aspect of the serial reaction time paradigm is the implicit nature of the learning effect: participants do not report that sequences are repeated during the task (Seger 1994). In the serial reaction time task Nissen and Bullemer (1987) investigated motor-sequence learning in KS patients. KS patients showed the same speeding curve for the repeated sequences, although they were slower than the controls and made more errors in the first blocks, suggesting that motor-sequence learning on this specific task is still spared in KS despite slower response times. Subsequently, Van Tilborg et al. (2011) showed comparable results as Nissen and Bullemer (1987), but found no evidence of less accurate response. Their study included 20 KS patients and 11 control subjects, making it the largest currently available study on motoric procedural learning in KS (Table 1).

**Table 1:** Summary of results of experimental studies on motoric procedural learning in Korsakoff’s syndrome

Author	Year	Sample	Task	Outcome
Cermak et al.	1973	9 KS	Pursuit rotor	KS patients showed intact acquisition of the pursuit rotor task, but their acquisition of the maze task was less pronounced than in HC and AC.
		9 ALC	Maze test	
		9 HC		
Brooks & Baddeley	1976	3 KS	Pursuit rotor	KS and EC patients showed intact acquisition of the pursuit rotor task. Their acquisition of the maze task was diminished compared to HC. Performance was retained in all groups after 1 week.
		2 ENC	Maze test	
		5 HC		
Heindel, et al.	1988	2 KS	Pursuit rotor	The KS, IN and AD patients showed preserved motor skill learning, while the patients with HD showed no evidence of learning.
		1 ANO		
		1 INF		
		10 HD		
		10 AD		
		10 HC		
McEntee et al.	1987	8 KS	Pursuit rotor	KS patients learned both tasks and their increase of performance related to dopaminergic activity.
			Mirror tracing	
Nissen & Bullemer	1987	6 KS	Serial reaction time	KS patients were slower and less accurate than HC, but learned the serial reaction time task.
		8 HC		
Nissen et al.	1989	7 KS	Serial reaction time	KS patients learned and maintained the serial reaction time tasks and maze task, but failed to accomplish the same amount of learning on the maze task. Performance was preserved after 1 week.
		8 ALC		
		7 HC	Maze task	
Van Tilborg et al.	2011	20 KS	Serial reaction time task	KS patients were slower than HC, but learned the serial reaction time task with the same amount of errors.
		11 HC		
Swinnen et al.	2005	11 KS	Arm coördination task	KS learned and maintained less than HC, but were better able to do so when feedback was provided.
		11 HC		

*KS* Korsakoff’s syndrome, *ALC* Alcoholics, *HC* Healthy Controls, *ENC* Encephalitic amnesia, *ANO* Anoxia induced amnesia, *INF* Cerebral infarction induced amnesia, *HD* Huntington’s disease

Nissen et al. (1989) applied the serial reaction time task and a maze task. The authors showed that the learned sequence in the serial reaction time task was retained over 1 week without training. The performance on the serial reaction time task was comparable in healthy controls, alcoholics, and KS patients, but performance on the maze task was deteriorated. The results on the maze tasks are more elaborately discussed below in the paragraph on spatial procedural learning. According to the authors, performance in the serial reaction time task is much more constrained than in unstructured tasks, such as the maze task. They argue that constraining the response selection is an essential element for motoric procedural learning in KS. This notion was later supported by Swinnen et al. (2005). These authors developed a task that required coordination of the forelimbs such that one forelimb was 90° out-of-phase with the other, which is not a regular motoric action in daily life and therefore requires practice. Eleven KS patients and eleven healthy controls practiced for 2 days, with and without feedback on their coordination. The KS patients were able to learn and maintain the coordination task in the feedback condition, but showed less learning when feedback was withheld. According to the authors, the key to motoric memory preservation in KS is that perceptual information is made available to drive the motoric action.

#### Summary

In brief, there is abundant evidence that patients with KS are able to learn motoric procedures, often at the same rate and level of performance as healthy controls. Initial studies on the pursuit rotor task that suggested that all forms of motoric procedural learning are intact in KS were later nuanced by findings on the serial reaction time task. In the serial reaction time task, KS patients were slower than the controls and made more errors in the first learning blocks, suggesting that motor-sequence learning is not fully intact but spared to a reasonable extent. In order to be able to exert effective coordination in a novel movement task, KS patients seem to require feedback to enhance procedural learning. The quality of the available evidence on motoric procedural learning is restricted due to the relatively small sample sizes and the lack of control participants in many studies. Nevertheless, it is important to note that even in experiments that showed hampered task performance in KS compared to healthy controls, still evidence was reported for spared motoric learning potential.

### Cognitive Procedural Learning in Korsakoff’s Syndrome

A discrepant form of procedural learning is cognitive procedural learning. Cognitive procedural learning involves the learning of a strategy or a procedure to perform a cognitive skill. In contrast to motoric procedural learning do these skills not necessarily require a motoric response, but rather require the mastering of a law or algorithm that requires cognitive reflection. One could argue that cognitive procedures do require more cognitive resources than motoric procedures leading to hampered task performance in KS.

#### Mirror Reading and Visuoperceptual Learning

A cornerstone paper by Cohen and Squire (1980) described cognitive procedural learning in the form of mirror reading. Mirror reading involves the procedural learning of a perceptual-verbal skill. Four KS patients, three patients with electroconvulsive therapy induced amnesia, one patient with acquired brain damage and six controls saw cards with three nouns reflected by a mirror. Subjects were asked to read the words out loud and press a button after finishing the trial. Half of the items were repeated throughout the experiment, the other half were not. There were five blocks of ten word triads on three consecutive days and one block after 13 weeks. Surprisingly, all subjects became quicker on nonrepeated trials and repeated trials, but the curve of acquisition was steeper for the repeated trials. In the group of patients with amnesia this effect was less pronounced than in controls, but it was still evident. The patients showed explicit memory problems for recognizing the presented words, but even after 13 weeks without training, the mirror-reading procedure was retained in KS as in healthy controls. The authors argue that there are clear-cut differences between the basal ganglia mediated nondeclarative learning and declarative learning. In two later studies, the findings of Cohen and Squire (1980) were replicated in eight KS patients (Martone et al. 1984) and one KS patient (Beaunieux et al. 1998). Both studies were case-controlled with ten healthy controls. These studies on mirror reading suggest that patients with KS are able to learn and maintain the ability to read mirrored words, but they require additional learning sessions compared to healthy controls and they do benefit less from word repetition. In a later study by Fama et al. (2006) four KS patients, nine alcoholic patients and twenty-one healthy controls were asked to identify two sets of incomplete pictures. Although patients became better in the identification of repeated stimuli, there was no transfer of learning to a different set of stimuli with a comparable level of difficulty, suggesting that learning appeared to occur at a stimulus level instead of a skill level.

#### Laws and Algorithms

In quite a different paradigm, Wood et al. (1982) showed the learning of a Fibonacci’s law by six patients. In this experiment patients had to predict numerical series based on the minimal amount of information that is need to predict Fibonacci’s series. In Fibonacci’s series, each next number is the sum of the two preceding numbers. There were three learning trials and two retention trials, one after 24 h and one after 17 weeks. Especially the latter trial was the longest interval between the test phase and the recall phase that was applied in any study on procedural learning. The KS patients showed a weaker learning curve than would normally expected by healthy control subjects, but strikingly the retention was just as good in the patients as in the controls. Moreover, there was a substantial gain in performance over learning sessions. A few years later, Charness et al. (1988) studied the learning of an algorithm to square two-digit numbers in KS. One patient and seven healthy controls were asked to learn the algorithm in seven sessions on seven separate days. The patient was able to actively apply the algorithm to square numbers at the same rate as healthy controls, but he was unable to state the algorithm when asked. He was also not able to deal with exceptions to the algorithm. The authors therefore argue that the algorithm for the KS patient could be applied in a compiled fashion: the first step leads directly to the execution of the next step. They state that the algorithm task is likely to be one of the most complex cognitive procedural skills with only the Tower of Hanoi tasks as being more difficult (Table 2).

**Table 2:** Summary of results of experimental studies on cognitive procedural learning in Korsakoff’s syndrome

Author	Year	Sample	Task	Outcome
Cohen & Squire	1980	4 KS 1 ABD 3 ECT 6 HC	Mirror reading	KS, ABD and ECT patients acquired the skill at an equivalent rate as HC and retained it for 3 months.
Martone et al.	1984	8 KS 10 HD 10 HC	Mirror reading	KS, but not HD patients acquired the skill at a normal rate, but KS patients did not recognize the words while HD patients did.
Beaunieux et al.	1998	1 KS 1 ALC 10 HC	Mirror reading Tower of Hanoi	Both cognitive skills were learned at the same rate in KS as in HC and preserved after 1.5 h.
Wood et al.	1982	6 KS	Fibonacci’s law	All patients showed substantial gain of performance that was somewhat maintained after 1 day and 17 weeks.
Fama et al.	2006	4 KS 9 ALC 6 HC	Gollin Incomplete Picture Test	KS, ALC and HC had comparable levels of perceptual learning after correction for visuospatial impairment. Retention was normal after 1 h, but lower after 1 day in the KS patients. There was no transfer of learning over sets of pictures in KS.
Butters et al.	1985	5 KS 1 TUM 1 ENC 15 HD 12 HC	Tower of Hanoi	KS, TUM, ENC and HD patients were impaired relative to HC. KS patients showed some evidence of learning.
Beaunieux et al.	2013	14 KS 15 ALC 15 HC	Tower of Toronto	10 KS were able to perform the task, but obtained lower results than both CS and AL.

*KS* Korsakoff’s syndrome, *ALC* Alcoholics, *HC* Healthy Controls, *ABD* acquired brain damage, *HD* Huntington’s disease, *ECT* Electroconvulsive therapy induced amnesia, *TUM* Brain Tumor, *ENC* Encephalitic amnesia

#### Tower of Hanoi/Tower of Toronto

The Tower of Hanoi task was first applied in KS by Butters et al. (1985). Six patients with amnesia (5 KS, 1 amnesia patient with a brain tumor), 15 patients with Huntington’s disease and 12 healthy controls participated in this experiment. In this Tower of Hanoi task subjects were asked to arrange five blocks according to size on one of three wooden pegs. They were not allowed to place a larger block on a smaller one. To solve the puzzle, the subjects had to move the blocks around with an optimal solution of 31 moves. Subjects were required to solve the task eight times on two consecutive days. The patients with amnesia and the patients with Huntington’s disease failed to show the same improvement over learning trials as healthy controls. The authors argue that two factors could have contributed to the lack of finding improvement in KS: the Tower of Hanoi could have been a cognitive procedure that was too complex for the patients with severe memory disorders. Moreover, the Tower of Hanoi requires more cognitive abilities than the cognitive procedure of solving the puzzle. It also requires identification, sequencing and strategies to ensure an efficient solution. Later, the results by Butters et al. (1985) were questioned by Beaunieux et al. (1998). A patient with KS and ten healthy controls were able to learn an easier version of the Tower of Hanoi with three discs (optimal solution in seven moves) in the same amount of time as healthy subjects over three trials. After an interval of one-and-a-half hour performance was maintained. The authors argued that while the task was somewhat easier than in the study by Butters et al. (1985), the procedure was clear for the patient and it was not contaminated with other cognitive functions such as executive functioning or episodic memory which are compromised in KS.A recent study by Beaunieux et al. (2013) aimed to disentangle the contributions of cognitive procedural learning, working memory, executive functioning and declarative memory in the acquisition of the Tower of Toronto task. Fourteen KS patients, fifteen chronic alcoholics without Korsakoff’s syndrome and fifteen controls performed the task with three pegs and four discs on four consecutive days. The KS patients made more errors and needed more time to solve the puzzle than the alcoholics and healthy controls, but learned to perform the task. According to the Adaptive Control of Thoughts Model (Anderson 1992), cognitive procedural learning occurs in three subsequent phases. Learning a new cognitive procedure requires highly controlled processes in the first phase, but less controlled process in the second and third phase (Beaunieux et al. 2006). The authors argue that specifically the first phase of cognitive procedural learning in KS requires more time than in healthy subjects. An important difference between the patients that showed intact procedural learning in the Tower of Toronto task and the patients that did not show procedural learning was the degree to which executive functioning was impaired.

### Spatial Procedural Learning in Korsakoff’s Syndrome

Spatial memory is vitally important in everyday life. Without this type of memory we would constantly get lost, loose our belongings and we would not be able to make plans to navigate to any place. Spatial memory requires both declarative and nondeclarative aspects. Spatial procedural learning ranges from mediation of sensorimotor acquisition up to associations between environmental stimuli and responses (e.g., turning left at a central cross-point during navigation) (Passot et al. 2012). Both processes are involved in route learning and the learning of visuo-spatial regularities. The Maze task, the Spatial Pattern Learning Task, the Implicit Contextual Cueing Task and multiple forms of route learning and navigation have been applied.

#### Maze Task

Already in the first experiment on procedural learning in KS, the maze task was adopted. In a typical maze task participants needed to find the exit of the maze with their index finger with four or six cross-points. Often the time to accomplish the task and the number of required turning points are measured as indices of task performance. Cermak et al. (1973) showed that patients were less able to learn the maze task than healthy controls. In their experiment, KS patients made more errors and required more time to complete the maze task than healthy controls. The authors argued that also the learning of a maze task could be verbally mediated, resulting in hampered task performance in KS. Later, Brooks and Baddeley (1976) showed complementary results to the Cermak study (1973). KS patients made substantially more errors than healthy controls on the maze task, but they were able to accomplish better performance over ten consecutive trials. Moreover, after 1 week, performance was retained to the same extent as in healthy controls. The results of this study suggested that KS patients were still able to learn a maze task and also maintain it for a prolonged period, but the amount of errors was higher in the patient group than in the control group. Also, Nissen et al. (1989) applied a tactual stylus maze task. In the tactual stylus maze task participants needed to perform two types of mazes over 35 trials. In the blocked maze the alleys were blocked by small pieces of Plexiglass, in the unblocked maze the alleys were not blocked. Both types of maze had ten choice-points and were therefore more difficult than applied in earlier studies on maze learning in KS. KS showed an initial decrease of completion time from around 80 s in the first trial to around 40 s in the fifth trial. In the last 30 trials of the experiment, healthy subjects and alcoholic controls further increased their performance, but KS patients failed to do so. After 1 week, performance was not statistically significantly different from the last trial in KS patients. The results of this study suggest that KS patients could learn and maintain the maze task to some extent, but failed to learn in later trials. According to the authors, performance in the maze task is unconstrained which makes errors likely to occur in KS.

#### Spatial Pattern Learning Task

In the Spatial Pattern Learning Task, participants are required to move a cursor to one out of four circles on a screen. The participant needs to move the cursor to the target stimulus that turns red. Van Tilborg et al. (2011) showed that twenty patients diagnosed with KS and fourteen controls were able to move faster to the target when a pattern was repeated. Nevertheless, KS patients made more errors and showed less search facilitation than controls, showing evidence for a decline in spatial procedural learning in patients with KS. A number of participants noted that the sequences were not random, which could possibly have affected the results of the experiment. The authors argue that a strong spatial response component is the primary factor that resulted in less pronounced learning for patients with KS than healthy controls.

#### Implicit Contextual Cueing

Implicit contextual cueing is the ability to acquire contextual information from our surroundings without conscious awareness. In a typical implicit contextual cueing experiment, subjects need to find a target stimulus (a T) among distractors (Ls) during visual search. Some of the configurations of stimuli are repeated during the experiment resulting in faster responses than for novel configurations, without subjects being aware of their repetition. In our study on implicit contextual cueing, patients with KS were slower in responding than the matched controls, but the Implicit Contextual Cueing effect was similar in both groups. This results suggests that KS patients were able to learn repetitions of spatial configurations (Oudman et al. 2011). Importantly, both patients and healthy controls were not able to recognise the spatial configurations, suggesting that the process of spatial learning was implicit. The results of our study extend the results of Postma et al. (2008) on conscious and unconscious object-location memory. In their experiment, 23 patients had poor (explicit) memory for object locations in their natural surroundings. Strikingly, they performed slightly better than the controls on unconscious spatial memory, showing that unconscious and conscious influences of spatial memory are functionally distinct (Table 3).

**Table 3:** Summary of results of experimental studies on spatial aspects of cognitive procedural learning in Korsakoff’s syndrome

Author	Year	Sample	Task	Outcome
Postma et al.	2008	23 KS 18 HC	Object-location memory	Using the process dissociation procedure it became clear that KS and HC showed comparable influence of unconscious memory during an object-location memory task. After 1 week influence of unconscious memory was not affected.
Oudman et al.	2011	18 KS 20 HC	Implicit Contextual Learning	KS patients showed intact ability to to find a target among a number of distractors during visual search after repetition and without conscious recollection.
Van Tilborg et al.	2012		Serial reaction time task Pattern learning task	Implicit motor learning occurred in both groups of participants on the serial reaction time task; however, on the Pattern Learning Task, the percentage of errors did not increase in the Korsakoff group in the random test phase, which is indicative of less implicit learning.
Kessels et al.	2007	10 KS	Route learning task	Both errorless learning and trial-and-error learning supported a route learning task.

*KS* Korsakoff’s syndrome, *ALC* Alcoholics, *HC* Healthy Controls, *ABD* acquired brain damage, *HD* Huntington’s disease, *ECT* Electroconvulsive therapy induced amnesia, *TUM* Brain Tumor, *ENC* Encephalitic amnesia

Route learning in KS was investigated by Kessels, van Loon, and Wester (2007). In their study, ten patients with KS had to walk two routes on the hospital terrain. In four sessions on four consecutive days the patients walked one of the routes. On one route, the experimenter asked the patient which way to go. The patient had to guess the correct answer, until the correct response was made. On the other route, the experimenter told the patient which way to go. Both errorless learning and trial-and-error learning resulted in equal spatial procedural learning. For the condition in which the patient was asked which way to go, participants showed a clear reduction of errors over the four consecutive learning sessions, suggesting that spatial procedural learning was preserved to some extent.

#### Summary

Although many studies demonstrate that patients with KS have some ability to learn cognitive procedures, the majority of studies show that performance in the learning phase is compromised compared to healthy controls. This deteriorated learning performance is, however, followed by a relatively preserved recall after a prolonged time for mirror reading (13-weeks in Cohen and Squire 1980) and learning the Fibonacci-law (17-weeks in Wood et al. 1982). The available research suggests that is likely that the complex tasks, such as in the Tower-Tasks, require multiple cognitive functions that are compromised in KS. Patients with KS are able to learn spatial regularities depending on the specific task at hand, but do not have maximum performance if the task requires multiple free response options without constraining or the instructions are normally verbally mediated such as in the frequently adopted maze task. Specifically in the acquisition-phase of a cognitive procedure, executive functions and elaborated working memory functioning are required. Nevertheless, the majority of research establishes that cognitive procedural learning is at least partially spared and well-preserved in KS.

## Interim Discussion

Remarkably, all the of the papers reviewed here have given evidence of procedural learning potential in KS, despite the large variety of studies that have been reviewed. However, if we look at the level of learning potential, there are discrepancies between paradigms and studies. For example, the KS patients showed the same amount of learning as healthy controls in the pursuit rotor paradigm, a specific instance of motor skill learning (Cermak et al. 1973; Brooks and Baddeley 1976; Heindel et al. 1988). On the opposite, there was much less procedural learning on the four disc Tower task, an instance of cognitive procedural learning, for patients with KS compared to healthy controls (Beaunieux et al. 1998, 2013). This discrepancy between both outcomes is, however, likely based on task complexity rather than the type of procedural learning, since also task performance on the motoric procedural Serial Reaction Time Task and movement feedback task was hampered in KS (Nissen et al. 1989; Swinnen et al. 2005). In the next paragraph we discuss whether and how memory rehabilitation techniques can facilitate learning potential in KS.

## Rehabilitation of Procedural Memory in Korsakoff’s Syndrome

Since patients with KS can learn new procedures it is worthwhile to establish how memory rehabilitation techniques could facilitate procedural learning and memory in KS. Memory aids and assistive technology capitalize on intact aspects of procedural learning to support acquisition of new procedures (Wilson 2009). Such interventions can both be compensatory or supportive to procedural learning. Since both are relevant to successful memory rehabilitation in Korsakoff’s syndrome, both types of interventions are reviewed in the second part of this manuscript. It is important to note that in the past decade the knowledge how to apply memory rehabilitation techniques to support procedural memory has increased to some extent, but the body of knowledge is still in its infancy. The available evidence concerns memory aids and interventions based on errorless learning. Both topics will be discussed separately in this paragraph.

### Memory Aids: The Role of Assistive Technologies

The view that KS is a static condition that does not permit further recovery is a commonly held one (Smith and Hilman 1999). Fortunately, the number of papers that disregard this idea is growing and there is nowadays accumulating evidence that rehabilitation of memory is possible for KS (Svanberg and Evans 2013; Horton et al. 2014). In the recent years numerous case-studies and group-studies have been performed to investigate memory rehabilitation in KS. Rehabilitation for patients with KS tends to focus on two strategies that are sometimes combined in the intervention: the deployment of external memory aids and the use of Errorless Learning techniques (Kopelman et al. 2009). Currently, there are six case studies available that investigated the use of memory aids in KS. The first case study that investigated the use of an external memory aid to facilitate residual memory in KS was performed by Davies and Binks (1983). In one KS patient, prompt cards and leaflets were used by the experimenter and the wife of the patient in order to reduce the memory demands for the patient. It was essential for the patient that his wife would keep the prompt cards and leaflets up to date, but the patient was helped by the external memory aids. Associative cues at storage and retrieval could boost successful retrieval of information relevant for the KS patient to be more autonomous in daily life.

A study that incorporated traditional assistive technology in a holistic approach was performed by de Fatima Alves Monteiro et al. (2011). The authors described a 25-week neuropsychological rehabilitation program for a patient with KS. A weekly cognitive training session was accompanied with the use of assistive technology. The patient was learned to use notes, schedules, a week-program and a calendar over the course of the 25 weeks. Importantly, the patient in the case study was accompanied by two caregivers all day long for every day of the week. Both caregivers subjectively noted that memory failures in the patient were reduced by the intervention. In the behavioral descriptions of the caregivers it was verified that the patient resorted frequently to the appropriate use of memory aids. Here, assistive technology was helpful in a holistic rehabilitation approach, although the effectiveness of the memory aid was based on subjective reports rather than objective measures.

The first study on assistive technology that adopted an electronic aid was performed by Morgan et al. (1990). An electronic diary was used to improve the ability of a man with KS to attend therapy-groups. At the start of the project the patient was prompted by staff members until he had an attendance rate of 80 % in the 14th week of the experiment. At the 18th week the electronic diary was introduced that resulted in 100 % attendance to the groups, but this improvement was no longer significant since the average attendance rate was already relatively high and there was some variation in attendance between weeks. An important contribution of this study to the field on assistive technology in KS is that combinations of assistive technology and prompts by care members were applied that resulted in maximal attendance to an appointment.

More recently, de Joode et al. (2013) investigated the feasibility of the use of a personal digital assistant (PDA) to support prospective memory functioning in a patient with KS. The PDA in this study was more complex than a simple prompting device since the patient could enter his own appointments and add notes to the appointment. A virtue of this study, compared to the earlier studies on assistive technology in KS was that this study compared the use of a PDA with a simple memory watch and a time period without any assistive support. Three goals were formulated in this experiment, namely being on time, having a long-term goal (e.g., sending an email message at a certain time) and remembering to take medication. Due to absence of a number of valid observations it was difficult to discern a pattern in the outcome for the second and third goal. The main conclusion of the experiment was, however, that the PDA scored essentially the same as the memory watch on all three formulated goals, but both interventions had positive effects compared to the no aid condition. Both the patient and clinical staff members favored the PDA over the memory watch as an cognitive aid. Nine months after the experiment the patient was still primarily using the alarm function of the calendar for which he required supervision to keep the calendar up to date. The most recent study on assistive technology did not focus on prospective memory, but instead focused on remembering past events by use of assistive technology. In this study, a patient with KS was already able to make use of compensatory strategies through an agenda and prompts of family members (Svanberg and Evans 2013). A problem that still existed was that through her memory problem she had no longer evidence to support her sense of herself, resulting in mood problems. The SenseCam, a wearable, automatic camera was used to record five regular activities she performed. Originally, it was intended to have eight regular activities recorded, but the patient wanted to stop the experiment after some time. The images that were automatically made during the tasks were downloaded onto a laptop and the patient watched them each day. The subjective memory rating of the patient increased, but her mood did not change. The patient reported that “it was like watching someone else’s life”. The authors concluded that careful establishment of consistent support networks and normalization of the technology are useful to increase engagement and the SenseCam could be part of a holistic approach to memory rehabilitation in KS.

#### Summary

Over the past decades, the knowledge how to apply memory aids in KS has increased to some extent, but the body of research on assistive technology is still in its infancy. The available evidence from case studies suggests that assistive technology are most likely to have a positive effect when (1) the formulated goals are restricted, (2) there is much time available to guide the patient in the process of learning how to use the assistive technology and (3) when the use of assistive technology is combined with elaborated learning techniques or an element in a holistic approach. There is an absence of case-controlled research on the application of memory aids, but the available case studies suggest that KS patients still require assistance from a family member or therapist despite the successful use of a memory aid. It was quite striking that in at least two of the six case studies the intervention as planned was altered during the process because of the will of the patient or family members of the patient (de Joode et al. 2013; Svanberg and Evans 2013), which could be an inherent feature of rehabilitating a patient with KS.

### Errorless Learning

Errorless learning is a well-known learning technique that has been applied successfully in KS among other groups of patients with cognitive disorders (see Clare and Jones 2008 for a critical review). The technique was originally developed by Terrace (1963). The most essential element of this learning technique is that the patient is not allowed to make errors by eliminating guessing during the process of learning, to support the already comprised memory functioning. Errorless learning is specifically relevant for patients with KS, since it is thought to be dependent on intact implicit learning (Clare and Jones 2008).

**Fig. 2:**
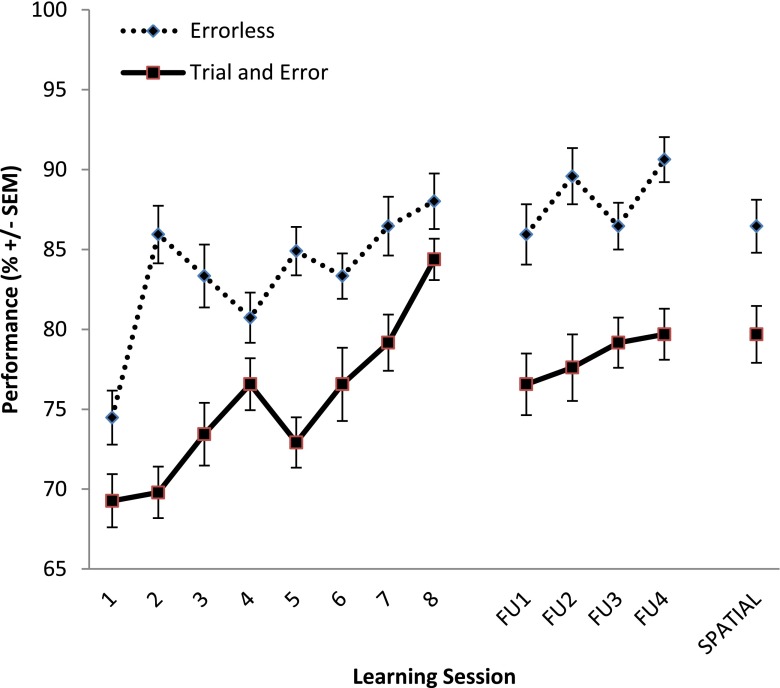
Performance on each learning session for Korsakoff’s syndrome patients in the errorless learning (*n* = 8) and trial and error learning (*n* = 8) condition. For total score comparisons, the total scores per session were adjusted to a 100-point scale. The sessions “FU1-FU4” represent a follow up after 4 weeks without training. The “Spatial” session was performed in a different spatial layout

To date, four studies on errorless learning in KS have been performed. The quality of the available evidence is mixed and none of the studies was case controlled. One of the first case studies on errorless learning in neurological patients included a description of a patient with KS (Wilson et al. 1994). In two experiments, the patient acquired the skill of entering information into an electronic memory aid. The errorless learning technique was more beneficial than the control condition (trial-and-error learning), suggesting that errorless learning was supportive to the residual memory of the KS patient. The first group studies on errorless learning in KS were performed by Komatsu et al. (2000). In their experiment, eight patients diagnosed with KS performed four study conditions to learn fictitious face-name relations. Two study conditions were based on the principle of errorless learning (paired associate and vanishing cue) and two study conditions were based on trial-and-error learning (target selection and initial letter). For all study conditions patients were trained twice a week for 2 weeks. In the paired associate condition, patients had to say the name of the face they saw on the screen which was displayed below the person. In the vanishing cues condition, the name of the face was also shown, but gradually was removed over five trials. In the target selection condition, patients needed to select the name of the person from five alternatives until they chose the correct one. In the initial letter condition, patients had to guess the name of the person they saw. After four guesses the correct name was displayed on the screen. The authors argued that both the vanishing cues condition and the initial letter condition required more effort than the paired associate and target selection condition. The results of the experiment showed that KS patients had most benefit from both errorless conditions (paired associate and vanishing cues), but a week after the experiment there was a floor effect for all conditions. The authors suggested that more learning trials were required and performed an additional experiment to test this hypothesis in four of the patients from the initial experiment. Although an increase of the trials did also increase the correct responses in the errorless conditions, no discrepancy between effortful and effortless errorless learning became clear. In a later study, errorless learning was also compared to trial-and-error learning in ten patients with KS to study route learning (Kessels, van Loon, and Wester 2007). In this within-subjects design both learning methods were effective to learn a novel route, but errorless learning was not better than trial-and-error learning. The authors suggested that the nature of the learned material (route learning) is likely to determine the benefit of errorless learning. An important aspect that could have contributed to the finding of the study was that the error rates were quite low in both experimental conditions and that more learning trials were needed to accomplish the beneficial effect of errorless learning. This study did not include a follow-up after a prolonged period. Recently, we performed a study on the acquisition of an activity of daily living, namely performing the laundry (Oudman et al. 2013). In our between-subject experiment, 16 patients with KS learned how to perform the laundry using a washing machine. Both errorless learning and trial-and-error learning were equally effective during the eight learning trials, but after a month without performing the laundry, errorless learning was beneficial compared to trial-and-error learning (see Fig. 2).

In the last session the spatial lay-out of the scene was changed. Performance in the errorless learning condition then deteriorated. The main finding of this study was, however, that errorless learning is a feasible technique for (re) learning an instrumental activity of daily living that could still be beneficial after a period without training.

#### Summary

There are currently four studies available on the effectiveness of errorless learning as a learning technique to learn new material or procedures in KS. Although the results are mixed, it is safe to say that errorless learning is beneficial compared to trial-and-error learning for some learning situations, such as learning names or procedural skills (Wilson et al. 1994; Komatsu et al. 2000; Oudman et al. 2013). Moreover, even if it is not more effective than trial-and-error learning it is effective (e.g., leading to a reduction of errors) to have a structured stepwise learning schedule (Kessels et al. 2007). Although it has been suggested that errorless learning is specifically effective for procedural learning, the beneficial effects of errorless learning are not only restricted to procedural tasks but also support name learning. Both verbal cueing and modeling seem relevant to the learning potential. The quality of the experiments on errorless learning is mixed and case-controlled studies are required. Importantly, the application of errorless learning always requires skilled therapist and is time-consuming. Although residual learning in KS is possible, errorless learning techniques are likely to result in fixed patterns of behavior that could not easily be manipulated (Oudman et al. 2013).

## Discussion

The aim of this review was to describe the available evidence on procedural learning, to highlight the advances in memory rehabilitation, and to discuss how memory rehabilitation techniques successfully may support procedural learning in KS. We evaluated 17 studies on procedural learning in KS and 9 studies on memory rehabilitation in KS, dating from 1976 to 2013. Based on the literature, there is substantial evidence that patients diagnosed with KS are able to learn procedural tasks and often even reach normal levels of task performance. Memory rehabilitation techniques for KS have been investigated in case studies and small-scale group studies, therefore the question how memory rehabilitation techniques can facilitate procedural learning in KS could only be answered tentatively. There is a specific hiatus concerning clinically relevant rehabilitation programs for KS. Based on our current review, we recommend that more rigorous, randomized, case-controlled studies are essential to develop a better understanding how memory rehabilitation can facilitate procedural learning in KS.

### What Could Hamper Procedural Learning in KS?

For certain procedural tasks, such as the pursuit rotor task or the serial reaction time task, procedural learning is fully preserved and maintained in KS after intensive practice (Cermak et al. 1973; Brooks and Baddeley 1976; Heindel et al. 1988; Nissen et al. 1989). For other procedural tasks, such as the Tower task and the maze task, learning performance is evident but reduced compared to healthy controls (Cermak et al. 1973; Brooks and Baddeley 1976; Nissen et al. 1989; Butters et al. 1985; Beaunieux et al. 2013). There are a number of interrelated explanations for protracted procedural learning in KS given in the literature. A first explanation relates to the cognitive preconditions for procedural learning that are not fulfilled in KS, a second explanation refers to the number of constrains during the procedural task at hand, and a final explanation focuses on the amount of feedback that is given after (un) successful procedural task performance. We will discuss each explanation briefly.Cermak et al. (1973) already found compromised procedural learning on the maze task and intact procedural learning on the pursuit rotor task. The authors explained that one could learn how to perform a maze task by remembering a sequence of left-right responses with the aid of verbal cues, but for the pursuit rotor-task this is not possible, probably resulting in a diminished learning potential on tasks that could be verbally mediated. This was the first study that put forward that for certain procedural tasks other cognitive processes, such as verbal memory, are of critical importance during the process of acquisition. In later studies, variations of this explanation for protracted procedural learning in KS have also been put forward to explain task performance on the Tower tasks (Butters et al. 1985; Beaunieux et al. 2006). For example, following Beaunieux et al. (2006) deficits in declarative memory and executive functioning could explain the hampered learning performance in KS in the initial phase of procedural learning. This observation was striking because the patient population diagnosed with KS presents itself with a relatively heterogeneous range of cognitive symptoms (Jacobson and Lishman 1987). More specifically, deficits regarding executive functioning, such as interference of information and perseverative responses are commonly but not necessarily present in KS, thereby contributing differentially to the procedural memory difficulties (see Brion et al. 2014 for a review). Variable executive deficits in KS could therefore results in diminished learning performance for KS patients as a group, but result in preserved learning in individual cases without the executive deficits.

A different explanation to clarify diminished learning potential in KS for some procedural tasks, is the amount of cueing that is given by the task. This explanation was first put forward to explain the difference in learning performance between successful learning in the serial reaction time task, but less successful learning in the maze task (Nissen et al. 1989). The authors suggested that procedural learning in KS is dependent on the extent to which the stimulus information *constrains* the response selection (i.e., a maze task gives no cues indicating which response should be made, until a response has been attempted, while the serial reaction time task constantly gives cues how to respond). Cueing is specifically relevant for procedural learning in KS, since patients with KS frequently show marked executive deficits (Oscar-Berman 2012). Task performance on a maze task, but also a relatively complex task as the Tower task is highly dependent on executive functioning. By cueing such tasks, for example through errorless learning, it is possible to maximally bypass executive functioning to learn the procedure correctly. A third explanation to clarify the discrepancy between successful and impaired learning of procedural tasks in KS is the amount of feedback that is given by the task. Swinnen et al. (2005) showed that patients with KS had a better learning potential when feedback was given on their performance in a task in which they needed to make an arm movement. Here, perceptual information was made available to drive the motoric action (Swinnen et al. 2005). To summarize, a combination of cognitive preconditions that have not been met by the patients with KS and task-dependent aspects such as a lack of constraints and feedback during the task all can hamper procedural learning in KS.

### New Routes for Using Memory Rehabilitation Techniques to Facilitate Procedural Learning in Korsakoff’s Syndrome

In the clinical literature, there has been a clear distinction between compensation and remediation of memory in memory rehabilitation. According to Rees et al. (2007), compensatory techniques, such as external or internal memory aids, for deficient memory functioning in patients with traumatic brain injury are currently the most promising forms of memory rehabilitation. Recent studies also show that remediation-based forms of therapy have the potency to increase memory functioning in patients with memory problems (Spreij et al. 2014). Unfortunately, many memory rehabilitation techniques have only been tested in patients with mild to moderate memory problems and not in patients with severe memory problems such as KS patients (see for example Cicerone et al. 2011). Moreover, it is currently unknown whether KS patients with extensive executive problems could successfully adopt memory aids to support their memory. In future research it would therefore be relevant to study the effects of compensatory techniques that decrease the amount of verbal mediation and increase the amount of cueing during a procedural task to support procedural learning. It would be relevant to specifically investigate the effectiveness of such techniques in groups of KS patients with mild and severe executive deficits separately. Recently developed technologies that could actively do so, also referred to as “smart objects” have not been adopted as memory rehabilitation aids in KS, while this could lead to amelioration of procedural learning based on other severe cognitive disorders (Stip and Rialle 2005). However, forms of memory rehabilitation that have been tested in patients with severe memory problems appear to be less successful compared to patients with less severe forms of amnesia (see for example Clare and Jones 2008).

Current state-of-the-art literature on memory rehabilitation in KS is solely based on uncontrolled case studies or small scale group studies (see Tables 4 and 5) and therefore warrants a rapid development of clinically relevant rehabilitation programs for KS. By formulating goals on forehand and restricting the learning procedure, the influence of deficits in declarative memory and executive functioning on procedural learning in KS can be reduced. Examples of such increased learning potential have been shown as the Tower of Hanoi task with three instead of four discs (Beaunieux et al. 1998), the maze task with blocked alleys (Nissen et al. 1989), and the successful introduction of prospective memory support devices (de Joode et al. 2013). Therefore, we suggest that a restriction of the formulated goals is recommended to facilitate procedural learning in KS. More recently, errorless learning has become increasingly popular as a teaching technique to guide successful procedural learning in multiple forms of severe cognitive problems, such as KS (Komatsu et al. 2000). In KS, errorless learning is more effective in learning face-name relationships and instrumental activities than learning with errors (Komatsu et al. 2000; Oudman et al. 2013). Recent findings suggest that procedural skills that are acquired through errorless learning are maintained over long periods and are relatively rapidly learned (Oudman et al. 2013). More research into the effectiveness of errorless learning for KS is of relevance, since the initial results of this type of interventions are promising.

**Table 4:** Summary of results of clinical studies on the application of memory aids in Korsakoff’s syndrome

Author	Year	Sample	Intervention	Outcome
Davies & Binks	1983	1 KS	Prompt cards and leaflets to reduce the memory demands for the patient	Cues at storage and retrieval boosted successful retrieval of information to be more autonomous in daily life.
Fatima Alves Monteiro et al.	2011	1 KS	A 25-week holistic neuropsychological rehabilitation program	The patient resorted frequently to the appropriate use of memory aids. No follow-up.
Morgan et al.	1990	1 KS	Electronic diary and verbal prompting to improve the ability to attend therapy-groups	Verbal prompting led to regular attendance of the therapy-groups, but the electronic diary could not further increase attendance.
de Joode et al.	2013	1 KS	Personal Digital Assistant (PDA) and memory watch to support being on time, having a long-term goal (e.g., sending an email message at a certain time) and remembering to take medication	PDA and memory watch supported being on time. Data on the other goals was missing. After the experiment, the patient stopped using the PDA and memory watch.
Svanberg & Evans	2013	1 KS	SenseCam, a wearable, automatic camera to record regular activities and support memory and mood	The experiment was stopped after 5 weeks. The patient reported increased subjective memory rating. Mood was not increased.

*KS* Korsakoff’s syndrome

**Table 5:** Summary of results of clinical studies on the application of errorless learning techniques in Korsakoff’s syndrome

Author	Year	Sample	Intervention	Outcome
Wilson et al.	1994	1 KS	Programming an electronic aid	The patient acquired the skill of entering information into an electronic memory aid. EL was more effective than TEL.
Komatsu et al.	2000	8 KS	Learning face-name relationships	KS patients learned face-name relationships over four consecutive learning sessions. The proportion of correctly learned names was higher in EL than in TEL.
Kessels et al.	2007	10 KS	Route learning	KS patients showed increased task performance on a route learning task over five consecutive sessions. EL and TEL were equally effective.
Oudman et al.	2013	16 KS	IADL learning	Both EL and TEL resulted in increased performance on the IADL over eight sessions, but in a follow-up after 4 weeks performance was only still elevated in TEL.

*KS* Korsakoff’s syndrome, *EL* Errorless learning, *TEL* Trial and Error Learning, *IADL* Instrumental Activity of Daily Living

## Conclusion

The aim of the present review was to give an overview of procedural learning and memory in KS to disentangle what processes are preserved. Also, we wanted to highlight current advances in assistive technology and memory rehabilitation to support procedural memory and learning in KS. The currently available evidence suggests that patients with KS are able to learn procedures, although the extent of learning is highly task dependent. The learning potential in KS can be ameliorated by recent advances in memory rehabilitation, but the state-of-the art interventions have only been investigated in small patient groups with heterogeneous cognitive and intellectual functioning. Patients with KS show maximum procedural learning potential when the task is minimally dependent on other cognitive domains than procedural learning, when feedback is given during the task and when the task itself is restricted in response options. We conclude that when memory rehabilitation is adjusted to the specific needs of KS patients, this will increase their ability to learn procedures and their normally compromised autonomy is enhanced.
